# Low-Density Neutrophils in Healthy Individuals Display a Mature Primed Phenotype

**DOI:** 10.3389/fimmu.2021.672520

**Published:** 2021-07-02

**Authors:** Carlos Blanco-Camarillo, Omar Rafael Alemán, Carlos Rosales

**Affiliations:** ^1^ Departamento de Inmunología, Instituto de Investigaciones Biomédicas, Universidad Nacional Autónoma de México, Mexico City, Mexico; ^2^ Posgrado en Ciencias Biológicas, Universidad Nacional Autónoma de México, Mexico City, Mexico

**Keywords:** neutrophil, inflammation, phagocytosis, neutrophil extracellular traps, reactive oxygen species

## Abstract

Neutrophils are the most abundant leukocytes in human peripheral blood, comprising about 70% of all leukocytes. They are regarded as the first line of defense of the innate immune system, but neutrophils have also the ability of regulating the adaptive immune response. Recently, However, multiple phenotypes and functional states of neutrophils have been reported, particularly in inflammation, autoimmunity, and cancer. One possible subtype of neutrophils, the so-called low-density neutrophils (LDN) is found among mononuclear cells (MNC), monocytes and lymphocytes, after separating the leukocytes from blood by density gradient centrifugation. LDN increase in numbers during several pathological conditions. However, LDN present in healthy conditions have not been investigated further. Therefore, in order to confirm the presence of LDN in blood of healthy individuals and to explore some of their cellular functions, neutrophils and MNC were isolated by density gradient centrifugation. Purified neutrophils were further characterized by multicolor flow cytometry (FACS) and then, using the same FACS parameters cells in the MNC fraction were analyzed. Within the MNC, LDN were consistently found. These LDN had a normal mature neutrophil morphology and displayed a CD10^+^, CD11b^+^, CD14^low^, CD15^high^, CD16b^high^, CD62L^+^, CD66b^+^, and CXCR4^+^ phenotype. These LDN had an enhanced reactive oxygen species (ROS) production and increased phagocytic capacity and were able to produce neutrophil extracellular traps (NET) similarly to neutrophils. These data confirm the presence of a small number of LDN is blood of healthy individuals and suggest that these LDN represent mature cells with a primed phenotype.

## Introduction

Neutrophils are the most abundant leukocytes in human peripheral blood, comprising about 70% of all leukocytes (1, 2). They are the first cells to arrive in large numbers to sites of inflammation or infection and therefore they are regarded as the first line of defense of the innate immune system (3, 4). Once they arrive at affected tissues, neutrophils reveal multiple antimicrobial functions including phagocytosis (5, 6), degranulation (7, 8), and formation of neutrophil extracellular traps (NET) (9). In a classical view, the antimicrobial activity of neutrophils was believed to be the only purpose for these leukocytes. However, in recent years this view has changed. It is now evident that neutrophils have not only effector functions in the innate immune response, but also have the ability of regulating the adaptive immune response (10–12). In addition, neutrophils have always been thought as homogenous cells with predetermined responses. Nonetheless, recently neutrophils with multiple phenotypes and functional states have been reported particularly in inflammation, autoimmunity and cancer (13), suggesting the existence of neutrophil heterogeneity (14–18). Among the several possible subtypes of neutrophils, the so-called low-density neutrophils (LDN) have caught much attention because they appear in larger numbers in several pathological conditions (19, 20).

These LDN have been found as a result of the method for purifying neutrophils from blood. Typically, leukocytes are separated through density gradient centrifugation (21, 22). Blood is placed on top of a density medium such as Ficoll-Paque, and after centrifugation, neutrophils sediment on top of red blood cells, separated from mononuclear cells (monocytes and lymphocytes), which for having more buoyancy are found in the upper part of the tube (low-density fraction) at the interface between the plasma and the Ficoll-Paque layers (**Supplemental Figure 1**). Among the mononuclear cells (MNC), cells with neutrophil morphology are also found. Thus, the name low-density neutrophils (LDN) has been used to describe them. Even though, LDN were reported a long time ago as a “contaminant” of MNC in patients with systemic lupus erythematosus (SLE), or rheumatoid arthritis (23), LDN have been more recently reported to be associated with cancer (24, 25), juvenile-onset SLE (26), SLE (27), inflammatory diseases, including sepsis (28), psoriasis (29), asthma (30), juvenile idiopathic arthritis (31), pyogenic arthritis, pyoderma gangrenosum and acne (PAPA) syndrome (32), anti-neutrophil cytoplasmic antibody (ANCA)-associated vasculitis (33), and several infections including HIV (34), *Plasmodium vivax* (35) and *Mycobacterium tuberculosis* (36). In addition, LDN have also been reported to be elevated in asthmatic horses (37). Therefore, conditions of immunosuppression and chronic inflammation seem to provoke the appearance of LDN.

Based on these reports, it has been postulated that LDN are a subtype of neutrophils that augment in blood as the severity of the disease increases, but that LDN are not present in healthy conditions (20). However, a close examination shows that most reports on LDN also mention the presence of LDN in healthy control individuals, with a frequency varying from 2 to 10% of the total MNC (23, 25–27, 29–32, 34–37). In most instances, these LDN were not investigated further. Thus, the presence of LDN in blood of healthy individuals remains unclear. In order to confirm the presence of LDN in blood of healthy people and to explore some of their cellular functions, neutrophils and MNC were isolated by density gradient centrifugation. Purified neutrophils were further characterized by multicolor flow cytometry (FACS) and then, using the same FACS parameters cells in the MNC fraction were analyzed. Within the MNC, LDN were consistently found. These LDN had a normal mature neutrophil morphology and displayed a CD10^+^, CD11b^+^, CD14^low^, CD15^high^, CD16b^high^, CD62L^+^, CD66b^+^, and CXCR4^+^ phenotype. In addition, these LDN had an enhanced reactive oxygen species (ROS) production and increased phagocytic capacity. These LDN could also produce NET similarly to neutrophils. These data confirm the presence of a small number of LDN in blood of healthy individuals and suggest that these LDN represent mature cells with a primed phenotype.

## Materials And Methods

### Reagents

Dextran T500 was from Pharmacosmos A/S (Holbaek, Denmark). Ficoll-Paque™ Plus, density 1.077 g/ml (catalog number 17-1440-03) was from GE Healthcare Bio-Sciences AB (Uppsala, Sweden). Bovine serum albumin (BSA) was from F. Hoffmann-La Roche Ltd. (Mannheim, Germany). Dihydrorhodamine123 (DHR-123) a ROS indicator (catalog number AS-85711), was from Anaspec, Inc (Fremont, CA, USA). Fetal bovine serum (FBS) was from ByProductos SA de CV (Guadalajara, Jalisco, Mexico) and the RPMI-1640 medium was from Gibco^®^, Invitrogen (Grand Island, NY, USA). DAPI, a cell-permeable DNA-binding dye (catalog number 268298) was from Calbiochem/EMD Millipore (Billerica, MA). SYTOX^®^ Green, a cell-impermeable DNA binding dye (catalog number S-7020) and MitoSOX™ Red, a mitochondrial superoxide indicator (catalog number M36008), were from Molecular Probes, Inc. (Eugene, OR). Fluorescent carboxylated latex beads (4.5 µm in diameter) (catalog number 16592) were from Polysciences (Warrington, PA, USA). Phorbol 12-myristate 13-acetate (PMA), a PKC activator (catalog number P8139) and all other chemicals were from Sigma Aldrich (St. Louis, MO, USA).

The following antibodies were used: anti-human Fc*γ*RIIa (CD32a) monoclonal antibody IV.3 (38) (ATCC^®^ HB-217) was from American Type Culture Collection (Manassas, VA, USA). Anti-human Fc*γ*RIII (CD16) monoclonal antibody 3G8 (39) was donated by Dr. Eric J. Brown (University of California in San Francisco, San Francisco, CA, USA). PE anti-human CD10 mouse IgG1 antibody (catalog number 312203), Alexa Fluor^®^ 488 anti-human CD11b mouse IgG1 antibody (catalog number 301317), APC/Cyanine7 anti-human CD14 mouse IgG1 antibody (catalog number 367107), PE/Cyanine5 anti-human CD15 mouse IgG1 antibody (catalog number 323013), APC anti-human CD62L mouse IgG1 antibody (catalog number 304809), Alexa Fluor^®^ 647 anti-human CD66b mouse IgM antibody (catalog number 305109), and APC anti-human CD184 (CXCR4) mouse IgG2a antibody (catalog number 306509) were from BioLegend^®^ (San Diego, CA, USA). The PE anti-human CD16b mouse IgG2a antibody (catalog number 550868) was from BD Pharmingen™ (BD Biosciences; San Diego, CA, USA).

### Blood Collection

Peripheral blood was collected from adult healthy volunteers (20 - 40 years old) following a protocol (in project 254434 from CONACyT, Mexico) approved by the Bioethics Committee at Instituto de Investigaciones Biomédicas – Universidad Nacional Autónoma de México (UNAM). Ten ml of blood collected with a syringe were immediately placed in a 15 ml tube containing heparin (25 IU/ml of blood), or with a DB Vacutainer^®^ (catalog number 368171) from Becton Dickinson Life Sciences (Franklin Lakes, NJ, USA) containing dipotassium salt of ethylenediaminetetraacetic acid (EDTA) (final concentration 5 mM) (40).

### Neutrophils

Neutrophils were purified exactly as previously described (22). Briefly, 10 ml of anti-coagulated blood were mixed with 2 ml of 6% dextran T500, mixed by inversion, and let to rest for 45 min to allow red blood cells to sediment. The leukocyte-rich plasma was layered on top of 5 ml Ficoll-Paque and centrifuged at 516 x g for 20 min at 4°C. After centrifugation, a layer of mononuclear cells (MNC), containing mainly monocytes and lymphocytes, is found at the interface of plasma and Ficoll-Paque. At the bottom of the tube, a pellet of purified neutrophils is found (21, 41). Both neutrophils and MNC were collected in separate tubes, washed once, resuspended in PBS, and kept on ice until used.

### Nuclear Staining and Microscopy

Purified cells, neutrophils or mononuclear cells (low-density fraction) were fixed with 1% paraformaldehyde and then incubated with 150 nM DAPI for 15 min. Cells were then observed with a fluorescence inverted microscope model IX-70 from Olympus (Center Valley, PA). Images were captured with an Evolution- VF Cooled Color camera from Media Cybernetics (Rockville,MD), and the computer program Q Capture pro 6.0 from QIMAGING Surrey (British Columbia, Canada). Images were processed with the computer program ImageJ v1.47 from The National Institutes of Health (Bethesda, MD, USA).

### Multicolor Flow Cytometry

Fluoresce staining of membrane molecules for analysis by flow cytometry was performed as described (42). Briefly, 1 x 10^6^ cells in a 1.5 ml Eppendorf tube, were labeled for 30 min at 4°C in the dark with the antibodies anti-CD10 (0.05 µg/ml), anti-CD11b (0.25 µg/ml), anti-CD14 (0.25 µg/ml), anti-CD15 (0.25 µg/ml), anti-CD16b (1/40 dilution), anti-CD62L (0.03 µg/ml), anti-CD66b (0.05 µg/ml), and anti-CXCR4 (0.1 µg/ml) in 100 µl of labeling buffer (PBS + 1% BSA + 0.1% NaN_3_). Cells were washed once with 1 ml of PBS and then fixed in 1% paraformaldehyde. Cells were analyzed in an Attune™ NxT flow cytometer (blue/red lasers) from Thermo Fisher Scientific (Carlsbad, CA, USA). Cells were gated by dot-plot analysis and 10,000 cells were acquired per sample. Data were analyzed with the FlowJo™ Software, version 10 (Becton Dickinson; 2019; Ashland, OR, USA).

### Measurement of Reactive Oxygen Species (ROS)

ROS production was assessed by detecting fluoresce changes in neutrophils loaded with dihydrorhodamine 123 (DHR-123). Cells (1 x 10^6^) were resuspended in 100 μl of 15 μM DHR-123 in PBS and incubated for 15 min at 37°C in the dark. Cells were washed with 1 ml PBS, and then resuspended in 100 μl of PBS containing 20 nM of PMA, and then incubated at 37°C in the dark for 50 min. Next, cells were washed in cold PBS and resuspended in cold 1% paraformaldehyde in PBS. Cells were stored cold in the dark until analyzed by flow cytometry using an Attune™ NxT flow cytometer from Thermo Fisher Scientific (Carlsbad, CA, USA) with the 485 nm (excitation) and 520 nm (emission) filters. Cells were gated by dot-plot analysis and 10,000 cells were acquired per sample. Data analysis was performed using the FlowJo™ Software, version 10 (Becton Dickinson; 2019; Ashland, OR, USA).

### Measurement of Mitochondrial Reactive Oxygen Species

Mitochondrial ROS production was assessed by detecting fluoresce changes in neutrophils loaded with MitoSox™ Red. Cells (1 x 10^6^) were resuspended in 250 μl of 5 μM MitoSox™ in PBS containing 1.5 mM Ca^2+^ and 1.5 mM Mg^2+^. Cells were incubated for 30 min at 37°C in the dark, with gentle agitations every 10 min. Cells were washed with 1 ml warm PBS/Ca^2+^/Mg^2+^, and then resuspended in 300 μl of PBS/Ca^2+^/Mg^2+^ for immediate analysis by flow cytometry using an Attune™ NxT flow cytometer from Thermo Fisher Scientific (Carlsbad, CA, USA) with the 485 nm (excitation) and 585 nm (emission) filters. Cells were gated by dot-plot analysis and 10,000 cells were acquired per sample. For stimulation, cells were resuspended in 5 μM MitoSox™ in PBS/Ca^2+^/Mg^2+^ containing 20 nM PMA, incubated at 37°C in the dark for 30 min, washed, and resuspended in 300 μl of PBS/Ca^2+^/Mg^2+^ for flow cytometry. Data analysis was performed using the FlowJo™ Software, version 10 (Becton Dickinson; 2019; Ashland, OR, USA).

### Cell Sorting

Mononuclear cells (low-density fraction) were resuspended in labelling buffer (PBS + 1% BSA + 0.1% NaN_3_) containing anti-CD16b (1/40 dilution) antibody and were incubated for 30 min at 4°C in the dark. Next, cells were washed once and resuspended in PBS at 2 x 10^6^ cell/ml. CD16b^+^ cells were sorted in a BD FACSAria™ cell sorter (Becton, Dickinson and Company; Franklin Lakes, NJ, USA) and recovered on heat-inactivated fetal bovine serum. Finally, cells were washed in PBS and resuspended in RPMI-1640 medium. Cells were stained with 150 nM DAPI for 15 min, and then observed with a fluorescence inverted microscope model IX-70 from Olympus (Center Valley, PA).

### NET Formation Assay

Neutrophils or cell-sorted low-density neutrophils (LDN) (1 x 10^5^) in 250 µl RPMI-1640 medium were added to each well of a 48-well tissue culture plate (Costar^®^ 3548; Corning Inc., Corning, NY, USA), and incubated in a humidified incubator with 5% CO_2_ at 37°C for 20 min. Then 50 µl of 120 nM PMA in PBS were added to each well. Plates were incubated in 5% CO_2_ at 37°C for 4 h. Next, 300 µl of 2% paraformaldehyde with 240 nM DAPI in PBS were gently added to each well. The plates were incubated for 60 min at room temperature. Finally, the plates were observed with a fluorescence inverted microscope model IX-70 from Olympus (Center Valley, PA). Images were captured as described above.

### Phagocytic Targets

Fluorescent beads were opsonized by following the manufacturer’s recommendations as previously described (43, 44). Briefly, beads were washed three times in 1 M boric acid, resuspended in 1 M boric acid containing 10 µg/ml of monoclonal antibody IV.3, and incubated overnight at 4°C in the dark with constant agitation. Next, beads were washed and resuspended in 10 mg/ml BSA in PBS at 1.25 x 10^8 ^ beads/ml.

### Phagocytosis Assay (Microscopy)

Phagocytosis assays were performed in the fluid phase as previously described (42, 44). Briefly, in a prechilled 1.5 ml Eppendorf tube, neutrophils (1x 10^6^ cells) in 100 µl of cold phagocytosis buffer (2 mM Ca^2+^, 1.5 mM Mg^2+^, 1% BSA in PBS) were mixed with 24 µl of IV.3-opsonized or non-opsonized fluorescent beads (3:1 bead:cell ratio). Cells were mixed gently and incubated at 37°C for 30 min. Next, tubes were placed into an ice/water bath for 2 min, and then centrifuged at 4000 rpm for 2 min in an Eppendorf microcentrifuge. Cells were resuspended in 100 µl of ice-cold trypsin- EDTA solution (0.05% trypsin, 1 mM EDTA in PBS) to detach uninternalized beads from the cells. After a 15-min incubation on ice, cells were washed with 500 µl of cold buffer MACS (0.5% BSA, 2 mM EDTA in PBS) and finally resuspended in 500 µl of cold 1% paraformaldehyde in PBS. Cells were then observed with a fluorescence inverted microscope model IX-70 from Olympus (Center Valley, PA), and images were captured as described above. The phagocytic index (PI) was defined as the number of ingested beads by 100 cells.

### Phagocytosis Assay of LDN by Flow Cytometry

To analyze the phagocytic capacity of LDN by flow cytometry, all mononuclear cells were mixed with opsonized fluorescent beads and then after phagocytosis cells were labelled with anti-CD14 and anti-CD15 antibodies to clearly identify LDN from monocytes. The phagocytosis assay of mononuclear cells (1x 10^6^) was performed in the fluid phase exactly the same as described above for purified neutrophils. After phagocytosis, cells were labeled for 30 min at 4°C in the dark with the antibodies anti-CD14 (0.25 µg/ml) and anti-CD15 (0.25 µg/ml), before being fixed with 1% paraformaldehyde in PBS. Cells were analyzed in an Attune™ NxT flow cytometer (blue/red lasers) from Thermo Fisher Scientific (Carlsbad, CA, USA). Cells were gated by dot-plot analysis and by selecting CD14^low^, CD15^high^ cells. Data were analyzed with the FlowJo™ Software, version 10 (Becton Dickinson; 2019; Ashland, OR, USA), and phagocytosis was reported as the percent of positive cells (cells internalizing at least one green fluorescent bead).

### Statistical Analysis

Quantitative data were expressed as mean ± standard error of mean (SEM). Single variable data were compared by paired-sample Student’s t-tests using the computer program KaleidaGraph^®^ version 4.5.2 for Mac (Synergy Software; Reading, PA, USA). Differences were considered statistically significant at a value p < 0.05.

## Results

### Low-Density Neutrophils Are Found in Blood of Healthy Individuals

**Figure 1:**
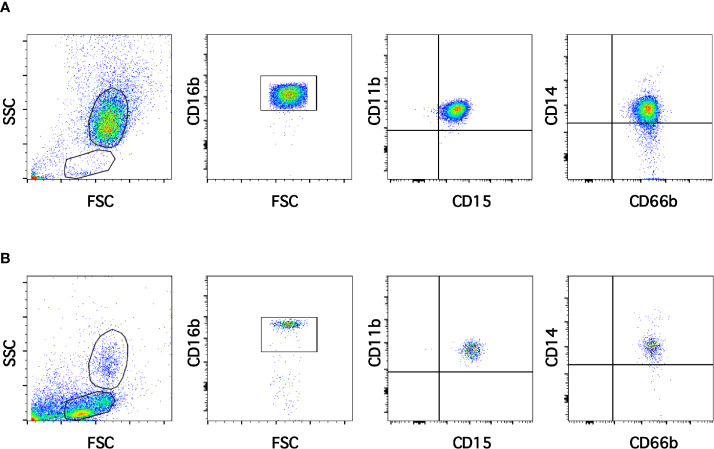
Low-density neutrophils are present in blood of healthy individuals. Leukocytes in blood from young healthy donors were separated by density gradient centrifugation into neutrophils **(A)** and mononuclear cells **(B)**. Purified neutrophils **(A)** were then analyzed by multicolor flow cytometry (FACS) for expression of CD16b, CD11b, CD15, CD14, and CD66b. Similarly, mononuclear cells **(B)** were analyzed with the same FACS parameters. A small number of cells with the same light scattering properties and the same membrane expression of molecules as neutrophils were found among MNC. Tracings are representative of more than 10 experiments with similar results.

**Figure 2:**
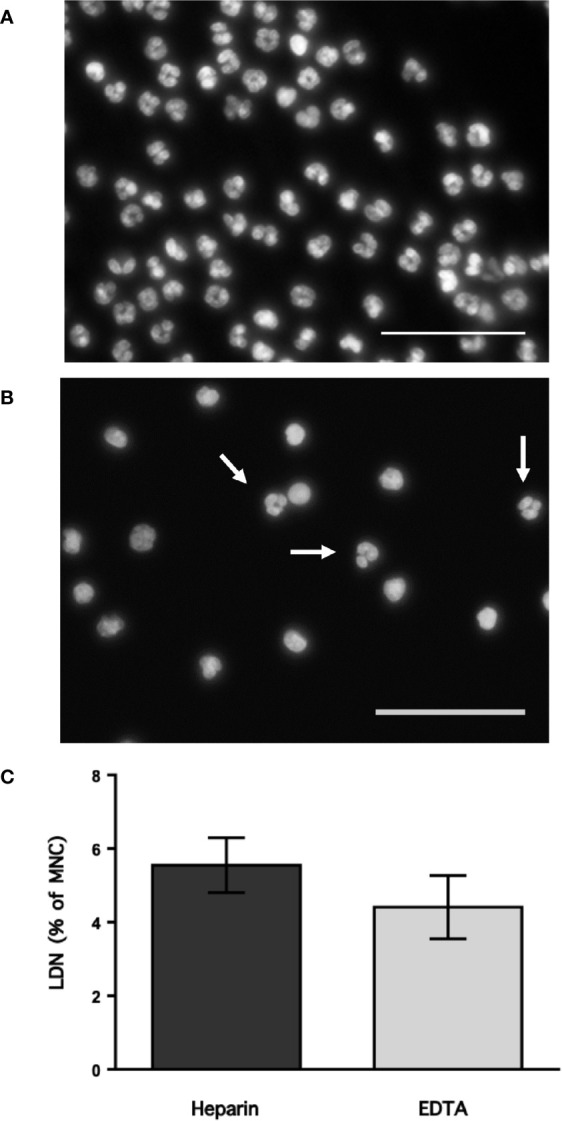
Low-density neutrophils have a mature neutrophil morphology. Blood from young healthy donors was anticoagulated with heparin or with EDTA before leukocytes were separated by density gradient centrifugation into neutrophils **(A)** and mononuclear cells **(B)**, and then staining with DAPI to visualize only DNA (nuclei) by fluorescence microscopy. Neutrophils **(A)** have typical multilobulated nuclei, while among MNC **(B)** several cells with the same nuclear morphology (white arrows) could be detected. Images are representative of 5 experiments with similar results. Scale bar represents 50 µm. **(C)** Low-density neutrophils (LDN) within mononuclear cells (MNC) were quantified by flow cytometry. Data are Mean ± SEM of five experiments. No difference (p = 0.347) was found between anticoagulants.

Neutrophils exhibit key functions for fighting infections, controlling inflammation, and regulating immune responses (11). In addition, in recent times several neutrophil subtypes have been reported in different settings, particularly inflammation, autoimmunity and cancer (18). One interesting subtype of neutrophils is the low-density neutrophils (LDN), which appear among mononuclear cells after separation by density gradient centrifugation (**Supplemental Figure 1**). These LDN are reported to increase in multiple pathological conditions (19). However, the presence of LDN in blood of healthy individuals remains unclear. In order confirm the presence of LDN in blood from healthy people, leukocytes were separated by density gradient centrifugation (22). Purified neutrophils, collected from the bottom of the tube were further analyzed by flow cytometry using fluorescent antibodies specific for molecules known to be expressed on neutrophils. Neutrophils were selected from side- and forward-scatter plots and then examined for expression of CD16b (FcγRIIIb) (45). Positive cells were next evaluated for CD11b and CD15 expression. Double positive cells were finally evaluated for CD14 and CD66b expression (**Supplemental Figure 2**). Purified neutrophils (purity > 95%) were a homogeneous cell population expressing CD16b (**Figure 1A**). These neutrophils were also positive for expression of CD11b, CD15, CD14, and CD66b (**Figure 1A**). This is the characteristic phenotype for normal mature neutrophils. Next, purified MNC were analyzed with the same FACS parameters established for neutrophils. The majority of MNC appeared outside the region for neutrophils in a dot plot (**Figure 1B**). However, it was evident that a good number of cells were present in the area selected for neutrophils. These cells, among the MNC population, also expressed the molecules CD16b, CD11b, CD15, CD14, and CD66b (**Figure 1B**). These cells had the same light-scattering properties and membrane surface markers as typical neutrophils, but had more buoyancy than neutrophils. Thus, among the MNC population from healthy individuals there were LDN. In addition, as expected purified neutrophils had a typical morphology with a multilobulated nucleus (**Figure 2A**). Among the MNC population from healthy individuals, cells with the typical morphology of neutrophils could also be detected (**Figure 2B**). These LDN were routinely found to represent around 5% of the total MNC (**Figure 2C**). Because, it has also been suggested that the presence of LDN in blood from healthy individuals could be induced by the use of different anticoagulants (20), the purification of leukocytes and FACS analysis were done with samples that included heparin or EDTA as anticoagulants. The properties and numbers of LDN in blood from healthy individuals did not vary independently of the anticoagulant used (**Figure 2C**). Together these data indicate that in blood of healthy individuals a small number of LDN are present. These LDN display a mature neutrophil morphology and expression of membrane molecules typical of normal neutrophils.

### Low-Density Neutrophils Display Higher Expression of CD15 and CD16b

**Figure 3:**
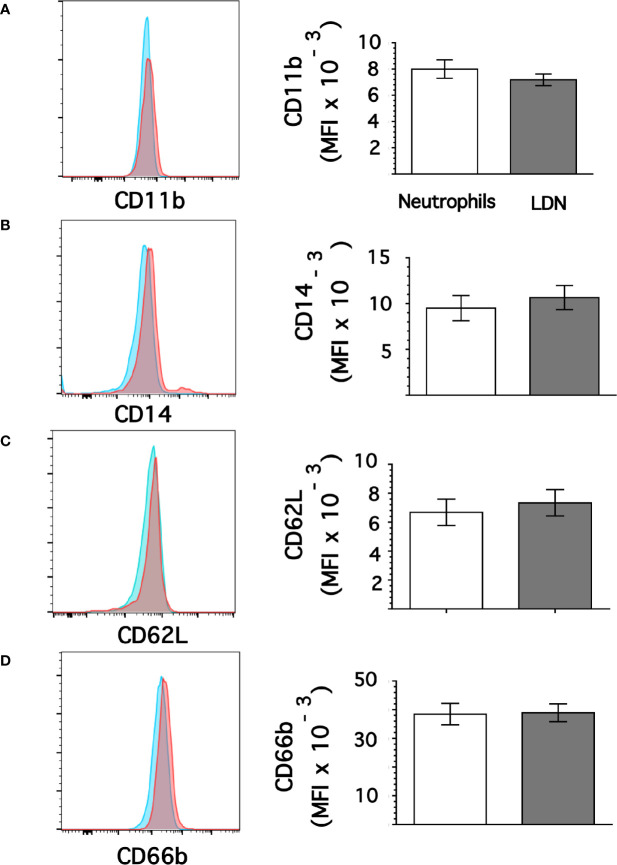
Low-density neutrophils display similar expression of CD11b, CD14, CD62L, and CD66b as neutrophils. Leukocytes in blood from young healthy donors were separated by density gradient centrifugation. Then, neutrophils (blue) or LDN (red) were analyzed by flow cytometry for membrane expression of CD11b **(A)**, CD14 **(B)**, CD62L **(C)**, CD66b **(D)**. Left panels are representative histograms of 11 experiments with similar results. Right panels show the Mean ± SEM of the mean fluorescence intensity (MFI) for each molecule.

**Figure 4:**
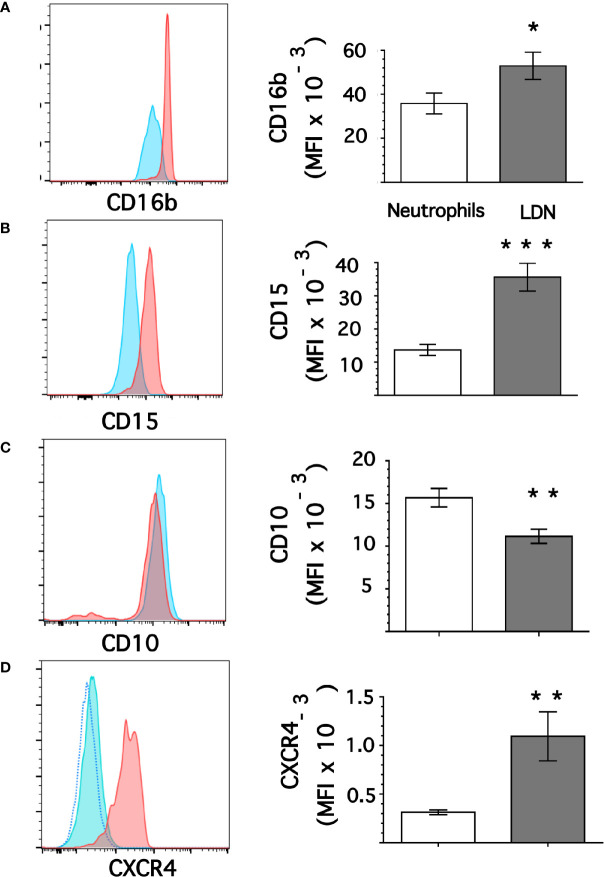
Low-density neutrophils display higher expression of CD15 and CD16b. Leukocytes in blood from young healthy donors were separated by density gradient centrifugation. Then, neutrophils (blue) or LDN (red) were analyzed by flow cytometry for membrane expression of CD16b **(A)**, CD15 **(B)**, CD10 **(C)**, CXCR4 **(D)**. Left panels are representative histograms of 11 experiments panel **(A)** or of 3 experiments panels **(B–D)** with similar results. The dotted line in panel **(D)** is for unlabeled (negative) cells. Right panels show the Mean ± SEM of the mean fluorescence intensity (MFI) for each molecule. Asterisks denote statistical differences between LDN and neutrophils (*p = 0.044, **p = 0.01, ***p = 0.001).

From flow cytometry dot plots (**Figure 1**) it seemed that membrane expression of some of the molecules was different between neutrophils and LDN. Histograms of membrane expression of each molecule indicated that levels of CD11b (**Figure 3A**), CD14 (**Figure 3B**), CD62L (**Figure 3C**), and CD66b (**Figure 3D**) were similar on neutrophils and on LDN. In contrast, CD16b (**Figure 4A**) and CD15 (**Figure 4B**) molecules were expressed at higher levels on LDN than on neutrophils. Also, the maturation marker CD10 was expressed on LDN (**Figure 4C**), although at slightly less level than neutrophils. A small fraction of LDN (14.7 ± 4.4%, mean ± SEM, n = 4) did not express CD10 (**Figure 4C**). In addition, the maturation marker CXCR4 (C-X-C chemokine receptor type 4) was also expressed on LDN (**Figure 4D**). This molecule was not expressed on neutrophils (**Figure 4D**). Therefore, the molecular pattern of these LDN was CD10^+^, CD11b^+^, CD14^low^, CD15^high^, CD16b^high^, CD62L^+^, CD66b^+^, and CXCR4^+^ and suggested that LDN in healthy individuals display a mature primed phenotype.

### Low-Density Neutrophils Produce More Reactive Oxygen Species Than Neutrophils

**Figure 5:**
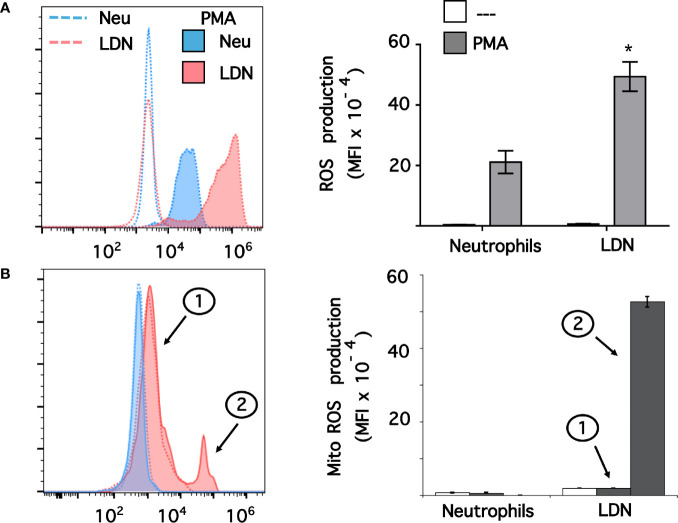
Low-density neutrophils produce more reactive oxygen species than neutrophils. **(A)** Reactive oxygen species (ROS) production was assessed in dihydrorhodamine 123-loaded cells by detecting fluoresce changes in flow cytometry. **(B)** Mitochondrial ROS (Mito ROS) production was also assessed in MitoSOX™ Red-loaded cells. Neutrophils (blue) or low-density neutrophils (LDN) (red) were left untreated (—), or were stimulated with 20 nM PMA (shaded histograms). The majority of LDN (peak 1) did not produced any mitochondrial ROS. A small fraction (13%) of LDN produced mitochondrial ROS after PMA stimulation (peak 2). Histograms (left panels) are representative of 9 separate experiments **(A)** or 3 separate experiments **(B)** with similar results. ROS production is indicated by the mean fluorescence intensity (MFI). Data (right panels) are the Mean ± SEM. Asterisks denote conditions that were statistically different between stimulated neutrophils and stimulated LDN (*p < 0.0003).

Data above suggested that LDN had a pre-activated (primed) phenotype. Thus, we explored ROS production by these LDN. Neutrophils or MNC were loaded with dihydrorhodamine 123, and then left untreated of stimulated with phorbol 12-myristate 13-acetate (PMA). Next, neutrophils or LDN were analyzed by flow cytometry (**Supplemental Figure 3**). All neutrophils stimulated with PMA produced, as expected, a good level of ROS (**Figure 5A**). Similarly, all LDN produced ROS after PMA stimulation (**Figure 5A**). Few LDN (13.6 ± 4.8%, mean ± SEM) produced ROS at a level similar to neutrophils. However, the majority of LDN (86.7 ± 2.3%, mean ± SEM, n = 4) produced an even more robust (twice as much) amount of ROS than neutrophils (**Figure 5A**). Clearly, these LDN were more responsive to stimulation and produced high levels of ROS. Because, LDN from SLE patients have been described to have increase mitochondrial ROS production (46), we also explored the capacity of LDN from healthy individuals to form mitochondrial ROS. Neutrophils stimulated with PMA did not produce mitochondrial ROS (**Figure 5B**). Similarly, nearly all LDN did not produce mitochondrial ROS (**Figure 5B**). However, a small fraction of LDN (13.5 ± 1.2%, mean ± SEM) was able to produce good amounts of mitochondrial ROS (**Figure 5B**). Therefore, LDN from healthy individuals produce more reactive oxygen species than neutrophils, and these ROS do not come from mitochondria.

### Low-Density Neutrophils Produce NET Similarly to Neutrophils

**Figure 6:**
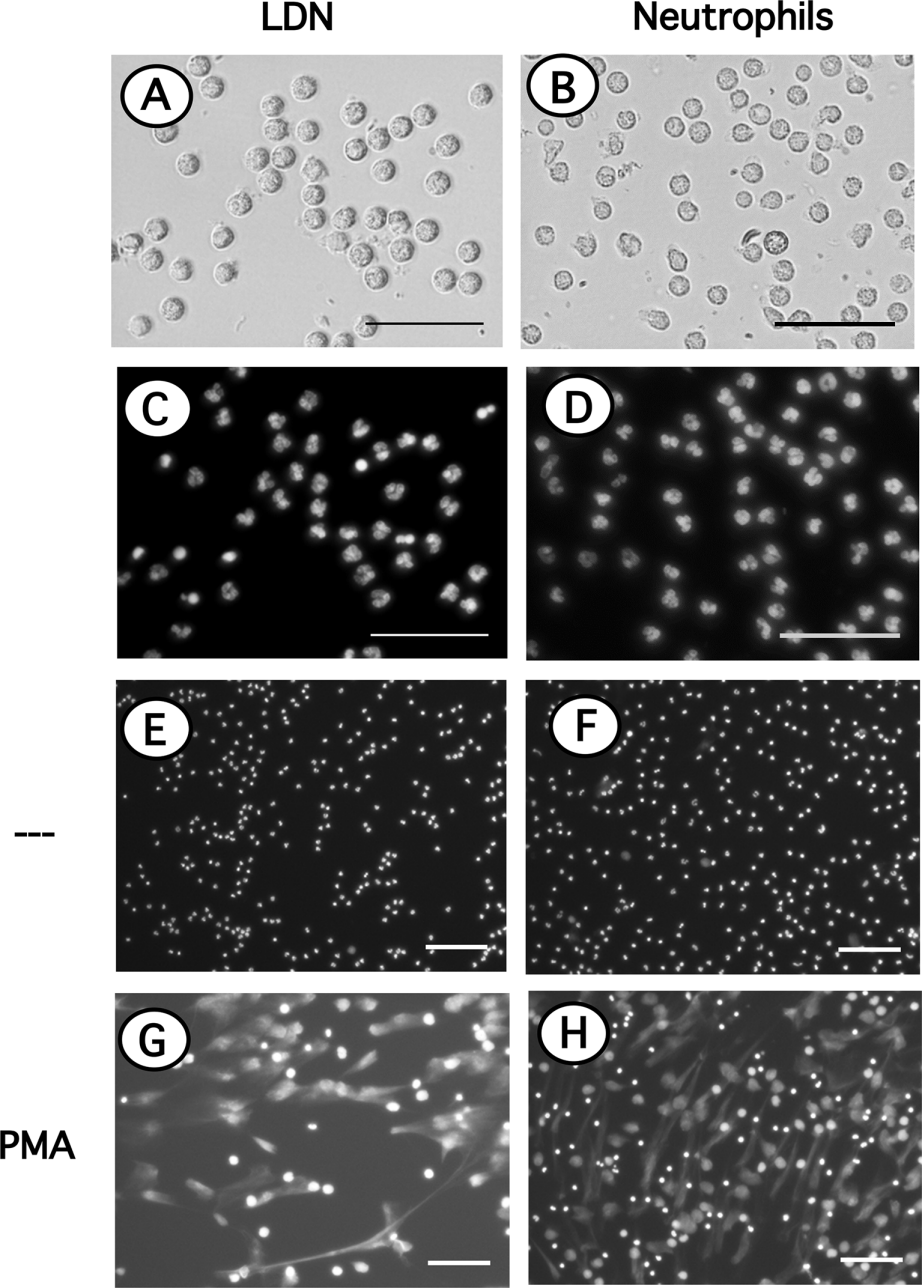
Neutrophils and LDN have similar size, nuclear morphology and produce similar NET. Neutrophils purified by density gradient centrifugation and low-density neutrophils (LDN) purified by cell sorting were stained with DAPI to visualize only DNA (nuclei) by fluorescence microscopy. LDN **(A)**; bright field) and neutrophils **(B)**; bright field) have similar size and nuclear morphology **(C, D)**. LDN **(E, G)** or neutrophils **(F, H)** were left untreated (—), or were stimulated with 20 nM PMA. After four hours cells were stained with DAPI to visualize neutrophil extracellular traps (NET). Images are representative of 2 experiments with similar results. Scale bar represents 50 µm **(A–D)**, or 100 µm **(E–H)**.

Since LDN seemed to be more reactive and it has been suggested that LDN in several pathological conditions are prone to release NET (32, 47, 48), the response of LDN to PMA to form NET was evaluated next. LDN are found among the more abundant MNC, thus in order to appreciate NET formation by LDN alone, these cells were first purified by cell sorting. Purified LDN (**Figure 6A**) showed a homogeneous population of cells with similar size to neutrophils (**Figure 6B**) and a mature neutrophil nucleus morphology (**Figure 6C**) similar to neutrophil nuclei (**Figure 6D**). Cell-sorted LDN (**Figure 6E**) or neutrophils (**Figure 6F**) were then stimulated by PMA. After four hours, both cell types have produced typical NET. No difference was noted between the time and amount of NET produced by either LDN (**Figure 6G**) or neutrophils (**Figure 6H**).

### Low-Density Neutrophils Have a Higher Phagocytic Capacity Than Neutrophils

**Figure 7:**
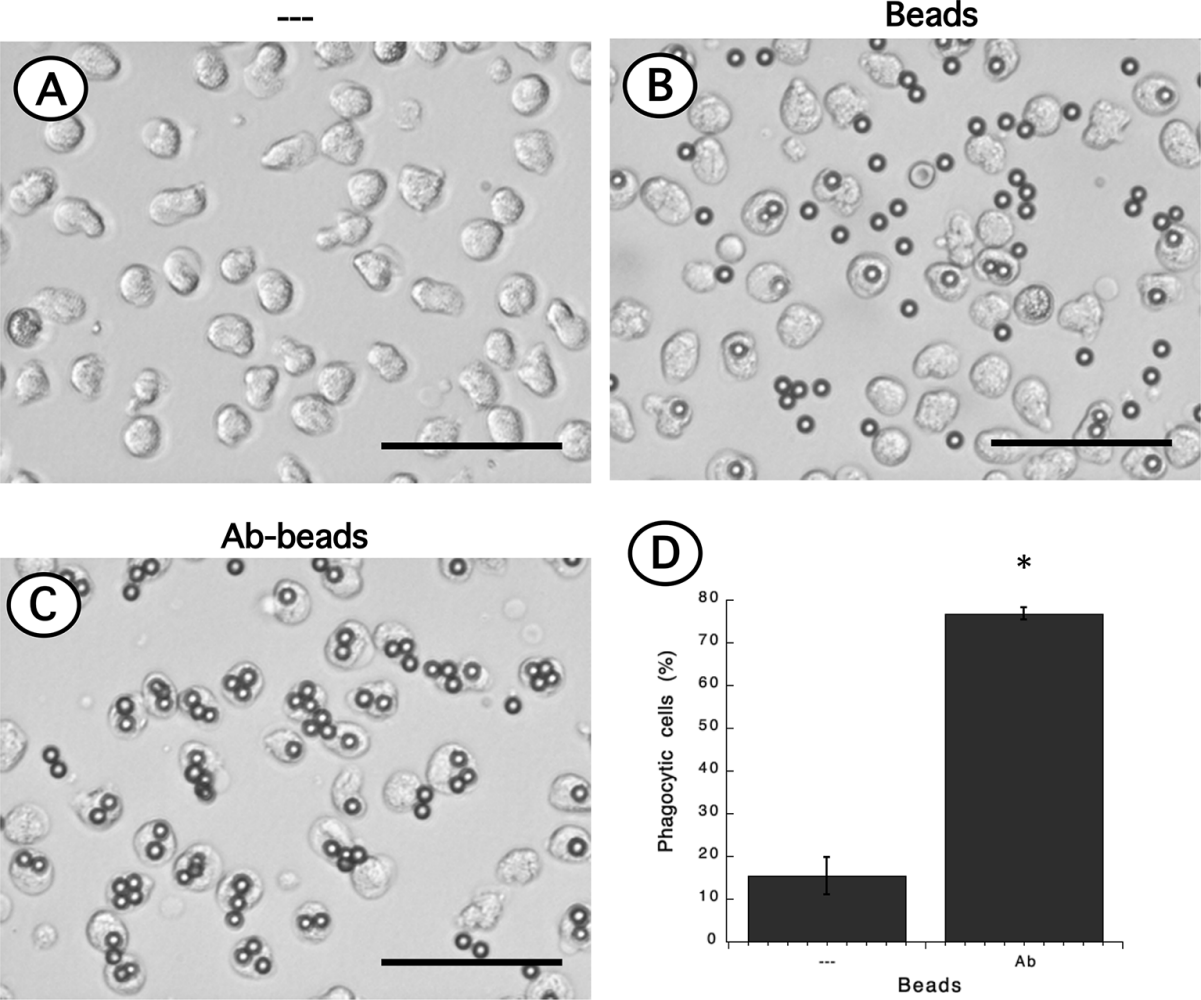
Phagocytosis of antibody-opsonized beads by neutrophils. Neutrophils alone **(A)** or mixed with non-opsonized latex beads **(B)**, or with antibody-opsonized latex beads (Ab-beads) **(C)** were incubated for 30 min at 37°C to assess phagocytosis. Images are representative of 5 experiments with similar results. Scale bar represents 50 µm. **(D)** Percentage of phagocytic neutrophils for non-opsonized (–), or antibody-opsonized (Ab) beads. Data are mean ± SEM of 5 separate experiments. Asterisk (*) denotes conditions that are statistically different between non-opsonized and antibody-opsonized beads (p < 0.0001).

**Figure 8:**
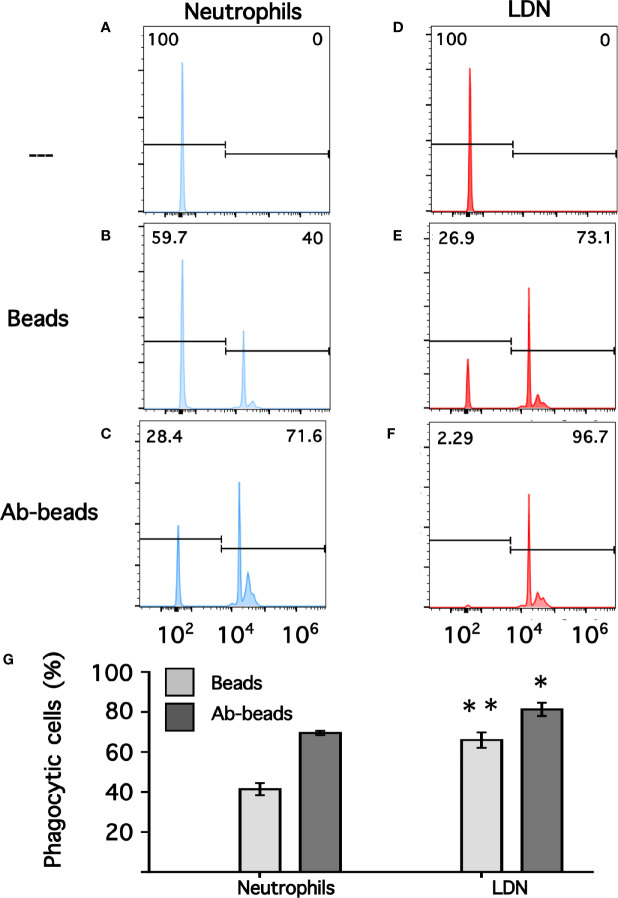
Phagocytosis by neutrophils and by low-density neutrophils. Neutrophils **(A–C)** or low-density neutrophils (LDN) **(D–F)** were left alone (—) or mixed with fluorescent non-opsonized latex beads (Beads), or with fluorescent antibody-opsonized latex beads (Ab-beads) and then incubated for 30 min at 37°C to assess phagocytosis. Increased fluorescence of cells represents ingested beads (phagocytosis). Histograms (blue for neutrophils, and red for LDN) are representative of 4 experiments with similar results. Graph **(G)** shows the percentage of phagocytic cells. Data are mean ± SEM of four independent experiments. Asterisks denote conditions that were statistically different between neutrophils and LDN. For non-opsonized beads (**p = 0.0024) and for antibody-opsonized beads (*p = 0.0142).

Phagocytosis is a fundamental antimicrobial function of normal mature neutrophils. Since LDN seemed to be more responsive than neutrophils, the phagocytic capacity of these cells was explored next. Neutrophils were mixed with antibody-opsonized latex beads in order to assess phagocytosis. About 15% of normal neutrophils were able to phagocytose non-opsonized beads (**Figure 7**); while almost 80% of neutrophils could ingest antibody-opsonized beads (**Figure 7**). In the same way, the phagocytic index (number of beads ingested by 100 neutrophils) was much higher for antibody-opsonized beads (140 ± 5.7, mean ± SEM, n = 5) than for non-opsonized beads (28.8 ± 11.3, mean ± SEM, n = 5). In an analogous way, total mononuclear cells were mixed with antibody-opsonized latex beads to assess phagocytosis. After a 30-min incubation at 37°C, there were few cells capable of ingesting beads (**Supplemental Figure 4**). These phagocytic cells could be monocytes or LDN. However, in this manner it was not possible to determine which type of cells the phagocytic cells were. Hence, phagocytosis by LDN was evaluated by flow cytometry. Mononuclear cells were mixed with fluorescent opsonized beads and after phagocytosis, the cells were labelled with antibodies against CD14 and CD15 to clearly separate monocytes from LDN. With this analysis, the fluorescence intensity of phagocytic cells increases only due to the fluoresce from ingested beads (**Supplemental Figure 5**). Neutrophils and LDN showed similar basal fluorescence in the absence of beads (**Figure 8**). When mixed with non-opsonized beads, around 40% of neutrophils were capable of ingesting beads, while around 70% of LDN were phagocytic (**Figure 8**). In the presence of antibody-opsonized beads, around 70% of neutrophils were capable of ingesting beads (**Figure 8**), whereas practically all LDN showed phagocytic capacity (**Figure 8**). These results showed that LDN from healthy individuals had an enhanced phagocytic capacity and suggest that they are more reactive neutrophils.

## Discussion

Neutrophils represent the most abundant leukocytes in blood and are considered the first line of defense during inflammation and infection (49–51). At damaged tissues neutrophils display several antimicrobial functions (4) including degranulation (7, 8), production of reactive oxygen species (ROS) (52, 53), phagocytosis (6) and the formation of neutrophil extracellular traps (NET) (9). In addition to these innate immune functions, neutrophils also participate in modulating the adaptive immune response (11). Also, neutrophils are traditionally considered to be homogenous cells with predetermined responses. However, recently neutrophils with multiple phenotypes and functional states have been reported in multiple diseases (13), suggesting the existence of neutrophil heterogeneity (14–18). One interesting group of neutrophils is the low-density neutrophils (LDN), which appear in larger numbers in several pathological conditions particularly immunosuppression, chronic inflammation, and cancer (19). Thus, it has been proposed that LDN are not present in healthy conditions, but rather they appear in blood as the severity of the disease increases (20). However, we noticed that most reports on LDN also mentioned the presence of LDN in healthy control individuals (23, 25–27, 29–32, 34–37, 54). Yet, in most instances these LDN were not investigated further. Now, in the present paper, we report that LDN were consistently found in blood of young healthy individuals. These LDN had a normal mature neutrophil morphology and displayed a CD10^+^, CD11b^+^, CD14^low^, CD15^high^, CD16b^high^, CD62L^+^, CD66b^+^, and CXCR4^+^ phenotype. In addition, these LDN had an enhanced ROS production and increased phagocytic capacity. These data confirm the presence of a small number of LDN is blood of healthy persons and suggest that these LDN represent mature primed cells.

LDN were discovered as a result of the method for purifying neutrophils from blood through density gradient centrifugation (21, 22). In this method, neutrophils sediment on top of red blood cells, separated from mononuclear cells (MNC), mainly monocytes and lymphocytes, which are found in the low-density fraction at the interface between the plasma and the Ficoll-Paque layers (**Supplemental Figure 1**). In this low-density fraction is where the LDN are found among MNC. For comparison purposes, the terms high-density neutrophils (HDN) or normal-density neutrophils (NDN) have been used to describe the “normal” neutrophils found in the lower (denser) part of the gradient. The term HDN is not accurate because it suggests that neutrophils have higher than normal density. We also think, as it has been proposed (55), that it should not be used while referring to neutrophils. The term NDN is more acceptable; however, it is also cumbersome. Therefore, we prefer the simple term “neutrophils” when mentioning these typical polymorphonuclear leukocytes, and the term “LDN” for the neutrophils with higher buoyancy found among MNC.

The origin of LDN remains elusive. Because both human and murine neutrophils change buoyancy after *in vitro* activation/degranulation by endotoxin-activated serum or by the chemotactic peptide fMLF (56), it is possible that degranulation leads to reduced density likely by the loss of granule contents, and stimulated neutrophils can now segregate together with MNC during density gradient centrifugation. Thus, it is believed that the presence of LDN in disease results from activation and degranulation of neutrophils *in vivo* (19, 35, 36). Support for this idea comes from the observation that LDN from patients with advanced adenocarcinoma had higher expression of activation/degranulation markers (57) such as CD11b (gelatinase granules) and CD66b (specific granules) than neutrophils from the same patient (58). But no direct evidence exist that neutrophils get activated *in vivo* and then degranulate turning into LDN. Furthermore, electron microscopy photographs of LDN show that they have similar granular content as neutrophils (59). In addition, another reason against degranulation being responsible for the change in cell density is the fact that most of the *in vitro* activated neutrophils return to their initial density after only a couple of hours (54, 60). It is improbable that neutrophils could regenerate their granules in such a short time. We also did not find evidence of degranulation in the LDN from healthy individuals, since the membrane expression levels of CD11b and CD66b were similar to those on neutrophils (**Figure 3**). A more likely process for changing the cell density of neutrophils is the change in cell volume (25) as a result of water uptake, regulated by aquaporin-9 (61). Therefore, it is possible that LDN are not *in vivo* activated/degranulated cells but rather primed neutrophils.

In addition to the activated (degranulated) phenotype suggested for LDN, several reports indicate that LDN are a heterogeneous population of cells comprised of both immature and mature neutrophils (24, 25, 62, 63). Immature (banded) neutrophils usually appear in circulation in response to acute and chronic inflammatory conditions. The response to inflammation leads to increased (emergency) granulopoiesis in the bone marrow, which releases many new cells into the blood including neutrophil progenitors (1, 64). Because these neutrophil progenitors have a lower density than neutrophils, it is not surprising that in systemic inflammatory conditions such as cancer or sepsis immature neutrophils are found among the MNC (65, 66). Also, when healthy volunteers were administered with lipopolysaccharide (LPS) banded neutrophils were recruited to the blood and many of them also appeared in the MNC fraction (54). However, in the case of healthy individuals, LDN do not seem to be immature neutrophils. These LDN express the maturation markers CD10 (67, 68) and CXCR4 (69) (**Figure 4**), clearly indicating that these LDN are mature neutrophils.

An important concern in the study of LDN is the fact that neutrophils from healthy individuals may become LDN as a result of inadequate blood handling and cell separation procedures (70). For example, neutrophil density gradually decreases when blood is stored at room temperature for more than 6 h before separation of leukocytes (71). Similarly, it is thought that some anticoagulants, such as heparin, may favor the appearance of LDN in blood of healthy people (20). Although we agree that blood handling is important for consistent results, we do not think that the LDN from healthy individuals found in many studies are always a consequence of inadequate blood handling. In our experiments, blood was processed immediately after collection (less than 5 min) and the purified cells were constantly kept at 4°C. In addition, the use of two different anticoagulants, namely heparin and EDTA, did not have any effect on the number of LDN found (**Figure 2**) nor on the expression of molecular markers. Thus, we believe that LDN are indeed present in low numbers in blood of healthy individuals. Our conclusion is similar to the conclusions reached in a study with asthmatic horses (37) and in two very recent reports on humans (54, 72).

In an effort to characterize LDN in different diseases other cellular features become relevant. Besides their physical properties, buoyancy and nuclear morphology, the expression of cell-surface proteins (markers) and cellular functions such as phagocytosis and NET formation have been fundamental (73). Unfortunately, there is not a single specific marker that can distinguish neutrophils from other leukocytes. Thus, a panel of several markers is used to define a neutrophil. Typically, human mature neutrophils are defined as CD14^−^, CD15^+^, CD16^+^, and CD66b^+^ (74, 75). However, together with the MNC another group of cells with immunosuppressive properties are also found. These cells are known as myeloid-derived suppressor cells (MDSC) and can be further divided into CD14^+^ monocytic (M-MDSC) and CD15^+^ granulocytic (G-MDSC) subsets (76, 77). For these cells a larger panel of markers is recommended, and the G-MDSC are defined as CD11b^+^, CD14^−^, CD15^+^, CD16^+^, CD66b^+^, and HLA-DR^-^ (70). Clearly, this set of markers also corresponds to neutrophils. Hence, some people think that LDN and G-MDSC are the same type of cells. For now, it is not possible to distinguish these two types of cells using only molecular markers and a functional assay to confirm their immunosuppressive properties becomes mandatory (78). In this report, we used a panel of antibodies against CD10, CD11b, CD14, CD15, CD16b, CD62L, CD66b, and CXCR4 to define a neutrophil. CD16 corresponds to the antibody receptor FcγRIII (79, 80). Two isoforms of this receptor exist, the FcγRIIIa (CD16a), which is a polypeptide-anchored molecule expressed on natural killer (NK) cells and macrophages and the FcγRIIIb (CD16b), which is a glycosylphosphatidylinositol (GPI)-anchored molecule expressed exclusively on human neutrophils (45, 81). Therefore, CD16b could be considered a specific marker for human neutrophils. However, the antibodies against CD16, such as monoclonal antibody 3G8 (39) cannot distinguish the two isoforms of FcγRIII. The new antibody used in this report, clone CLB-gran11.5, is specific for CD16b and as such it becomes an unambiguous marker for neutrophils. CD16b is polymorphic and the two codominant alleles are referred to as Neutrophil Antigen 1 (NA1) and Neutrophil Antigen 2 (NA2) (82). Clone CLB-gran11.5 reacts with neutrophils expressing the NA1 molecule. So, individuals expressing only the NA2 allele could not be detected with this antibody. Still, we found that indeed all mature neutrophils purified by density gradient centrifugation had the phenotype CD10^+^, CD11b^+^, CD14^low^, CD15^+^, CD16b^+^, CD62L^+^, CD66b^+,^ and CXCR4^+^ (**Figures 1**, **3**, **4**). Similarly, among the MNC a small number of cells presented exactly the same phenotype. Thus, these cells are truly mature LDN. In addition, an activated subset of human neutrophils with the phenotype CD16^bright^/CD62L^dim^ has been described in some pathological conditions such as systemic inflammation induced by endotoxin or by severe injury (66), *Staphylococcus aureus* infections (83), allergic rhinitis (84), and chronic lymphocytic leukemia (85). These CD62L^low^ neutrophils seem to have been activated in inflammation conditions and present different functions than normal neutrophils. The LDN present in healthy individuals have similar CD62L level of expression as neutrophils (**Figure 3**). Thus, these LDN do not appear to be activated cells but rather primed neutrophils.

The possible cellular functions of LDN are also a matter of intense controversy. As indicated above, reports from patients with cancer and chronic inflammation suggest that LDN are either immature neutrophils or G-MDSC with immunosuppressive properties (86, 87). Because, it is not possible to identify these cells molecularly, LDN should not be called immunosuppressive cells until they are shown to indeed have that function (78). Still, the issue is not that simple because already there are reports describing opposite effects of LDN on T-cells. In cancer, LDN can inhibit T-cell functions (88); but in SLE, LDN can activate T-cell responses (27). Thus, it seems that functions of LDN can vary among the different diseases where these cells are found. In the case of LDN from healthy individuals the information is even more scarce. In a previous report with asthmatic horses it was found that LDN from healthy animals were more sensitive to activation by PMA and had an increased capacity to produce NET, suggesting that LDN represent a population of primed mature cells (37). In the case of human LDN from healthy individuals, we also found that these cells were more responsive to PMA and produced more ROS than neutrophils (**Figure 5**). Human LDN also showed an enhanced phagocytic activity (**Figure 8**) and formed NET albeit at similar levels than neutrophils. We did not titrate the response of LDN to form NET in response to PMA. Since, Fc*γ*RIIIb (CD16b) is the receptor involved in NET formation by human neutrophils (43, 89) and LDN had higher expression of this receptor it is likely that LDN have indeed an increased capacity to produce NET. This will be explored in the future using direct stimulation of Fc*γ*RIIIb and a more physiological stimuli for NET formation, such as the parasite *Entamoeba hystolytica* (90, 91).

Our results are also in agreement with a very recent report on LDN from healthy people. In this study, LDN also did not show a difference in degranulation markers from neutrophils (54). The phagocytic activity against the bacteria *Staphylococcus aureus* was reported to be similar between LDN and neutrophils (54). This is in contrast to our results, which evaluated phagocytosis of antibody-opsonized particles. It is possible that basal phagocytosis is similar between LDN and neutrophils, but Fc receptor-mediated phagocytosis is enhanced in LDN from healthy individuals. Together, these data demonstrate that LDN are present in healthy individuals, both horses and humans, and these LDN have a different phenotype from neutrophils.

In conclusion, we have found that LDN are present in the blood of healthy individuals. These LDN had a normal mature neutrophil morphology and had an enhanced reactive oxygen species (ROS) production and increased phagocytic capacity. These LDN could also produce NET similarly to neutrophils. Our data suggest that these LDN represent mature primed cells.

## Data Availability Statement

The raw data supporting the conclusions of this article will be made available by the authors, without undue reservation.

## Ethics Statement

The studies involving human participants were reviewed and approved by Bioethics Committee at Instituto de Investigaciones Biomédicas – Universidad Nacional Autónoma de México (UNAM). The participants provided their written informed consent to participate in this study.

## Author Contributions

CB-C performed most of the experiments, analyzed data, and performed statistical analysis. ORA performed mitochondrial ROS experiments and analyzed data. CR designed the research, mentored other authors, prepared figures, organized the references, and wrote the paper. All authors contributed to the article and approved the submitted version.

## Funding

Research was supported by grant 254434 from Consejo Nacional de Ciencia y Tecnología (CONACyT), Mexico.

## Conflict of Interest

The authors declare that this research was conducted in the absence of any commercial or financial relationships that could be construed as a potential conflict of interest.
