# Corrigendum: Low-density neutrophils in healthy individuals display a mature primed phenotype

**DOI:** 10.3389/fimmu.2024.1477117

**Published:** 2024-09-02

**Authors:** Carlos Blanco-Camarillo, Omar Rafael Alemán, Carlos Rosales

**Affiliations:** ^1^ Departamento de Inmunología, Instituto de Investigaciones Biomédicas, Universidad Nacional Autónoma de México, Mexico City, Mexico; ^2^ Posgrado en Ciencias Biológicas, Universidad Nacional Autónoma de México, Mexico City, Mexico

**Keywords:** neutrophil, inflammation, phagocytosis, neutrophil extracellular traps, reactive oxygen species

In the published article, there was an error regarding the affiliations for Carlos Blanco-Camarillo. As well as having affiliation 1, he should also have “Posgrado en Ciencias Biológicas, Universidad Nacional Autónoma de México, Mexico City, Mexico”.

In the published article, there was an error. The graduate program for first author was not included in the **Acknowledgments** section.

A correction has been made to section **Acknowledgments**. This section previously stated:

“Authors thank Nancy Mora (Instituto de Investigaciones Biomédicas, UNAM, Mexico) for her excellent technical assistance. Authors also thank Idaira Guerrero and Michael Schnoor (CINVESTAV - IPN, Mexico) for providing the anti-CD10, anti-CD62L, and anti-CXCR4 antibodies; and to Verónica Garrido-Bazán and Jesús Aguirre-Linares (Instituto de Fisiología Celular, UNAM, Mexico) for providing the MitoSox™ Red reagent.”

The corrected sentence appears below:

“This article is part of the requirements for CBC for obtaining a Doctoral degree at the Posgrado en Ciencias Biológicas, UNAM. Authors thank Nancy Mora (Instituto de Investigaciones Biomédicas, UNAM, Mexico) for her excellent technical assistance. Authors also thank Idaira Guerrero and Michael Schnoor (CINVESTAV - IPN, Mexico) for providing the anti-CD10, anti-CD62L, and anti-CXCR4 antibodies; and to Verónica Garrido-Bazán and Jesús Aguirre-Linares (Instituto de Fisiología Celular, UNAM, Mexico) for providing the MitoSox™ Red reagent.”

The authors apologize for this error and state that this does not change the scientific conclusions of the article in any way. The original article has been updated.

