# Supplementary material for: Low-Density Neutrophils in Healthy Individuals Display a Mature Primed Phenotype
Source: Front Immunol. 2021 Jul 2;12:672520. doi: 10.3389/fimmu.2021.672520 (PMC8285102; doi:10.3389/fimmu.2021.672520)
Supplement: Supplementary file 1 [file DataSheet_1.pdf]

Supplemental Figure Legends

Supplemental Figure 1. *Purification method of human leukocytes from blood.* When human peripheral blood is placed over a Ficoll-Paque density gradient, after centrifugation neutrophils typically sediment on top of red blood cells (erythrocytes), whereas mononuclear cells (MNC), mainly monocytes and lymphocytes form a band at the interface between the plasma and the Ficoll-Paque layer. Among MNC, some neutrophils are also found. These cells are named low-density neutrophils (LDN).

Supplemental Figure 2. *Flow cytometry analysis scheme for neutrophils.* Density gradient centrifugation-purified human neutrophils were labeled with antibodies anti-CD11b, anti-CD14, anti-CD15, anti-CD16b, and anti-CD66b, and then analyzed by flow cytometry. Neutrophils (PMN) appearing as a homogeneous population of cells (R1 gate), were selected and examined for expression of CD16b. Positive ($CD16b^+$) cells (R2 gate) were next evaluated for CD11b and CD15 expression. Double positive cells (R3 gate) were finally evaluated for CD14 and CD66b expression. Double positive cells appear in the R4 gate. Mononuclear cells (MNC) appear in the side- (SSC) and forward- (FSC) scatter plot (dot-plot) analysis as a different cell population separated from neutrophils. Neutrophils were selected from side- (SSC) and forward- (FSC) scatter plots and then examined for expression of CD16b (FcγRIIIb)

Supplemental Figure 3. *Flow cytometry analysis for reactive oxygen species (ROS) production.* Neutrophils or mononuclear cells (MNC) were loaded with dihydrorhodamine 123, then stimulated with phorbol 12-myristate 13-acetate, and finally analyzed by flow cytometry. Neutrophils (blue) appear as a homogeneous population of cells (R1 gate) in side- (SSC) and forward- (FSC) scatter plots. Low-density neutrophils (LDN) (red) separate from MNC (green gate) and appear in the same R1 gate. Fluorescence intensity of cells in the R1 gate was compared to assess ROS production.

Supplemental Figure 4. *Phagocytosis of antibody-opsonized beads by mononuclear cells*. Total mononuclear cells were left alone (panel a) or mixed with non-opsonized latex beads (panel b), or with antibody-opsonized latex beads (Ab-beads) (panel c) and then were incubated for 30 min at 37 °C to assess phagocytosis. Images are representative of 5 experiments with similar results. Scale bar represents 50 μm. Among mononuclear cells there are few cells capable of ingesting Ab-beads. However, it is not possible in this assay to determine which type of cells the phagocytic cells are.

Supplemental Figure 5. *Flow cytometry analysis scheme for phagocytosis by flow cytometry.* Total mononuclear cells were incubated with antibody-opsonized fluorescent latex beads for 30 min at 37 °C to assess phagocytosis. After phagocytosis cells were labelled with anti-CD14 and anti-CD15 antibodies to clearly differentiate low-density neutrophils from monocytes. Cells and beads can be easily separated by flow cytometry in a side- (SSC) and forward- (FSC) scatter plot (dot-plot). Among mononuclear cells (MNC) some cells separate in the region where neutrophils are found (R1 gate). Cells in R1 were further analyzed for CD14 and CD15 expression. $CD14^+$, $CD15^-$ cells correspond to monocytes (Mono), while $CD14^-$, $CD15^+$ cells correspond to low-density neutrophils (LDN). The fluorescence intensity from ingested beads associated to LDN was evaluated in histograms, where an increase in fluorescence (shift to the right) within the M2 marker represents the phagocytic cells.

Figure S1

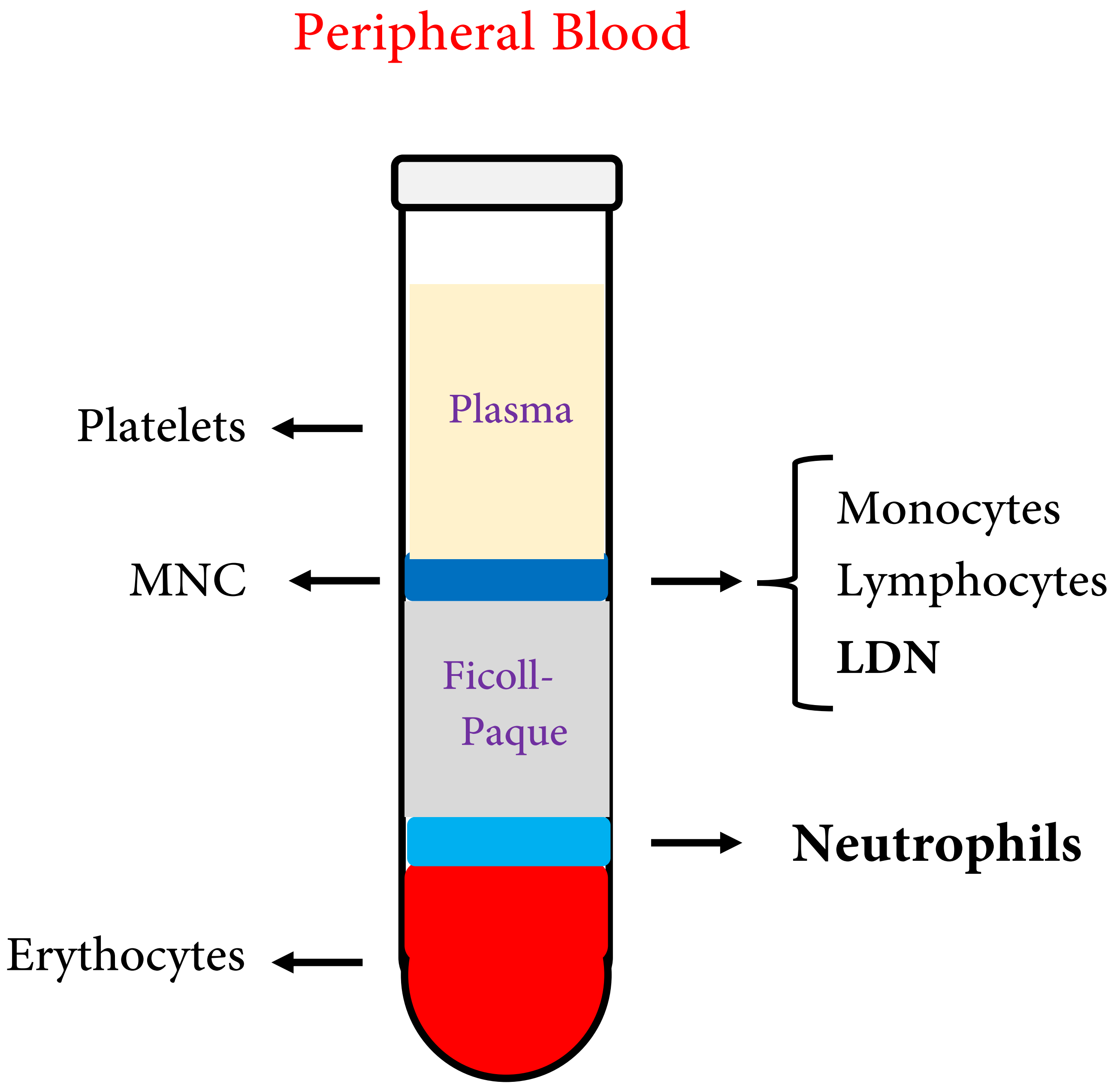
Peripheral Blood
Platelets
Plasma
MNC
Monocytes
Lymphocytes
LDN
Ficoll-
Paque
Neutrophils
Erythocytes

Figure S2

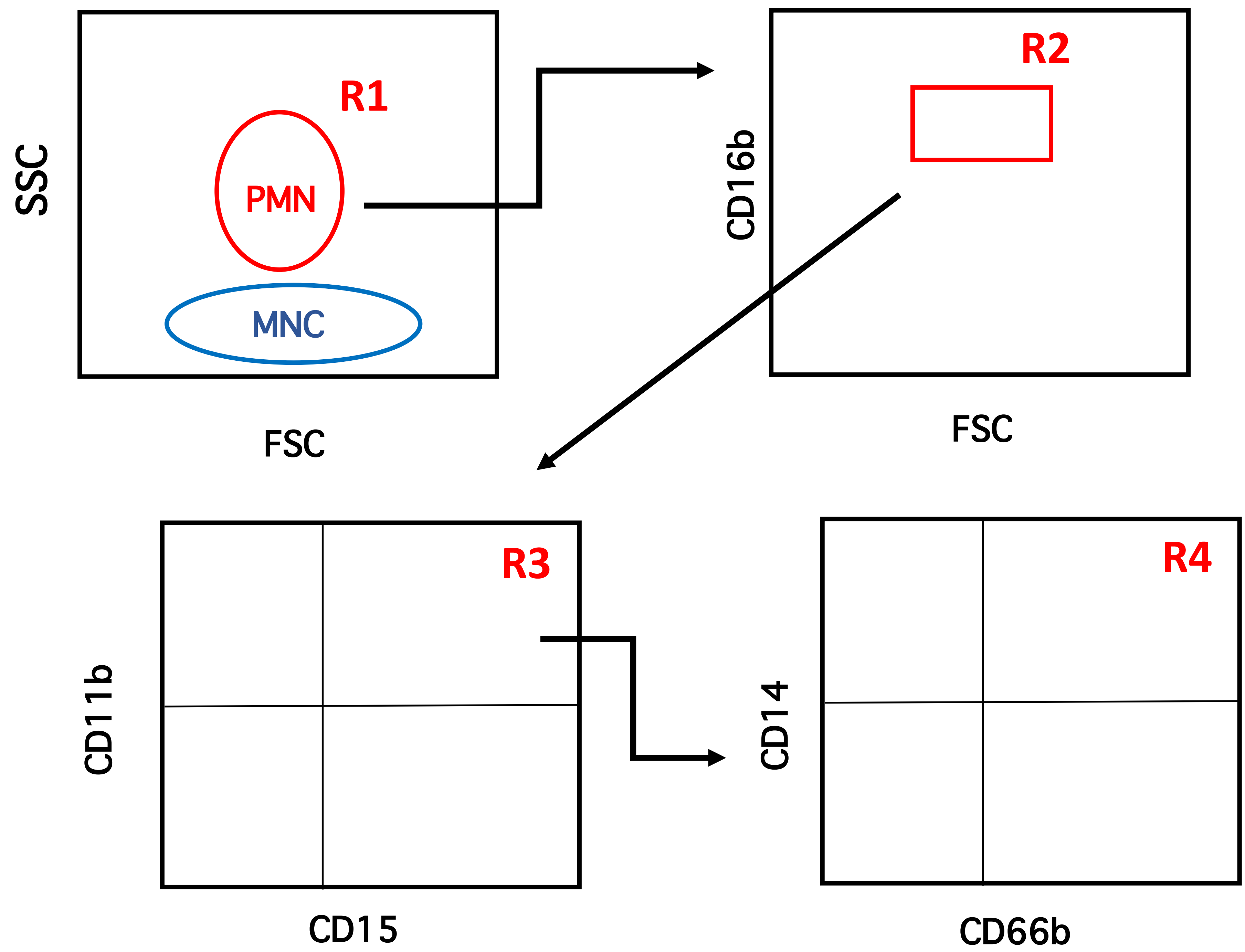

Figure S3

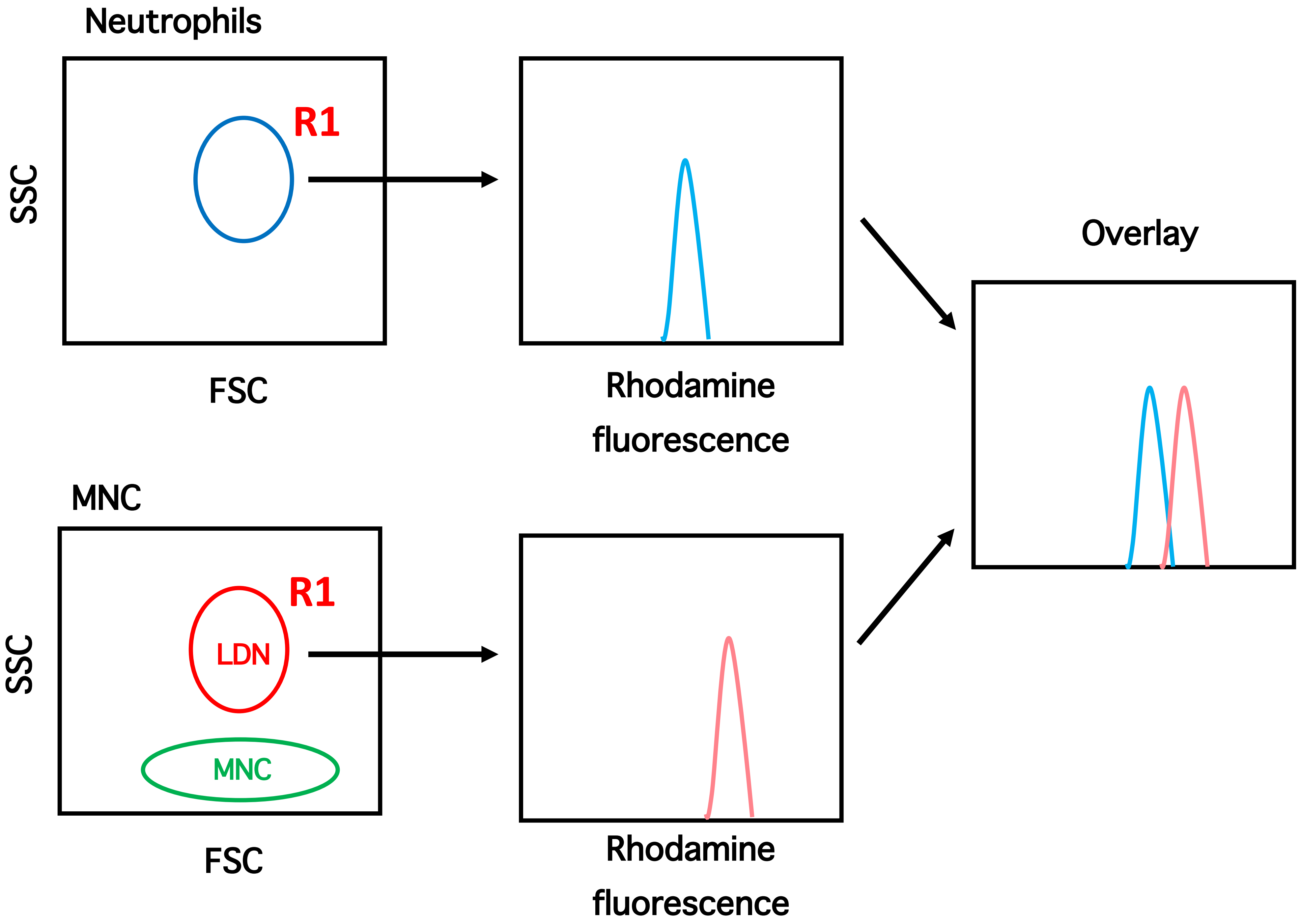

Neutrophils
R1
SSC
FSC
Rhodamine
fluorescence
Overlay
MNC
R1
LDN
MNC
SSC
FSC
Rhodamine
fluorescence

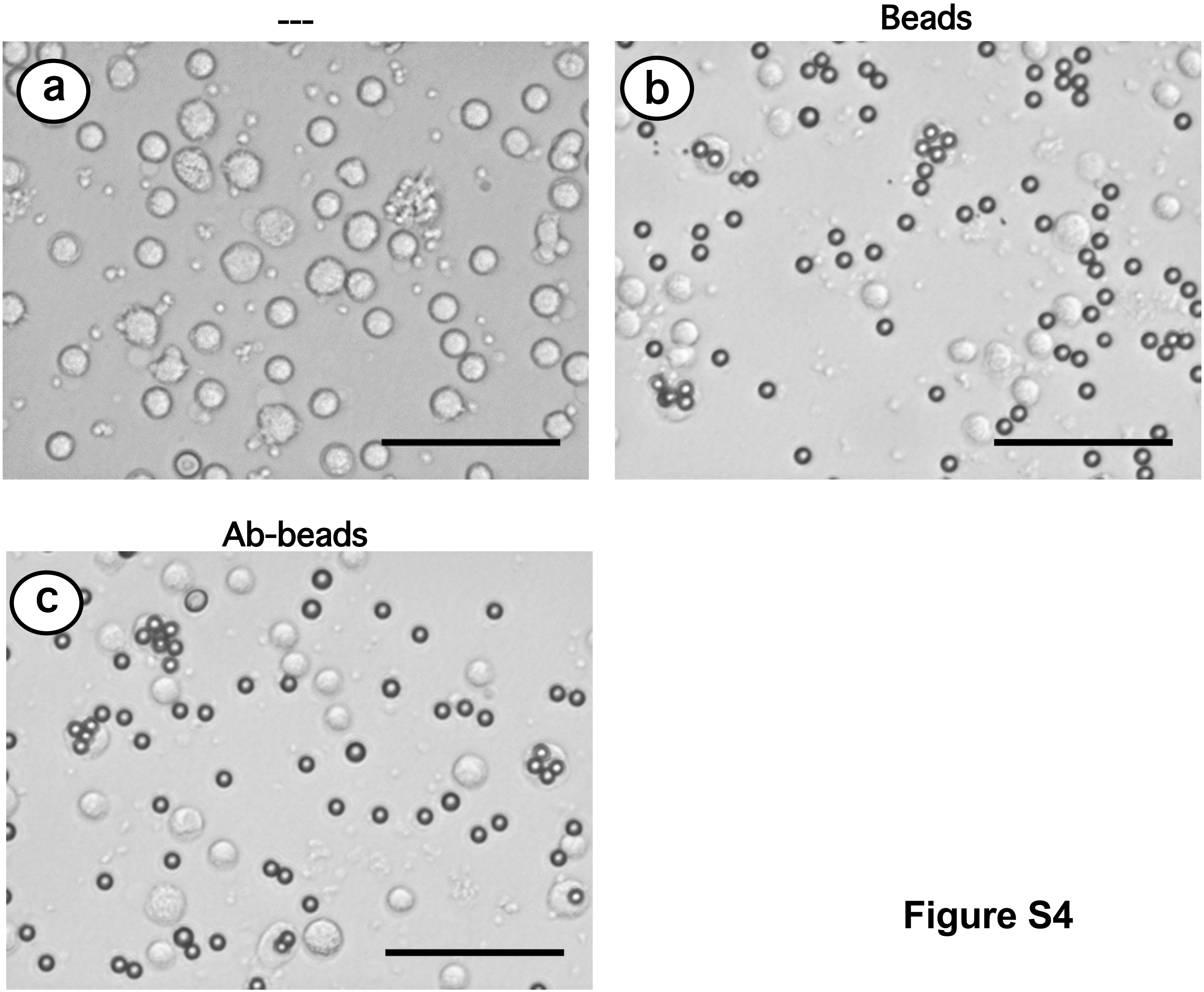

Figure S4

Figure S5

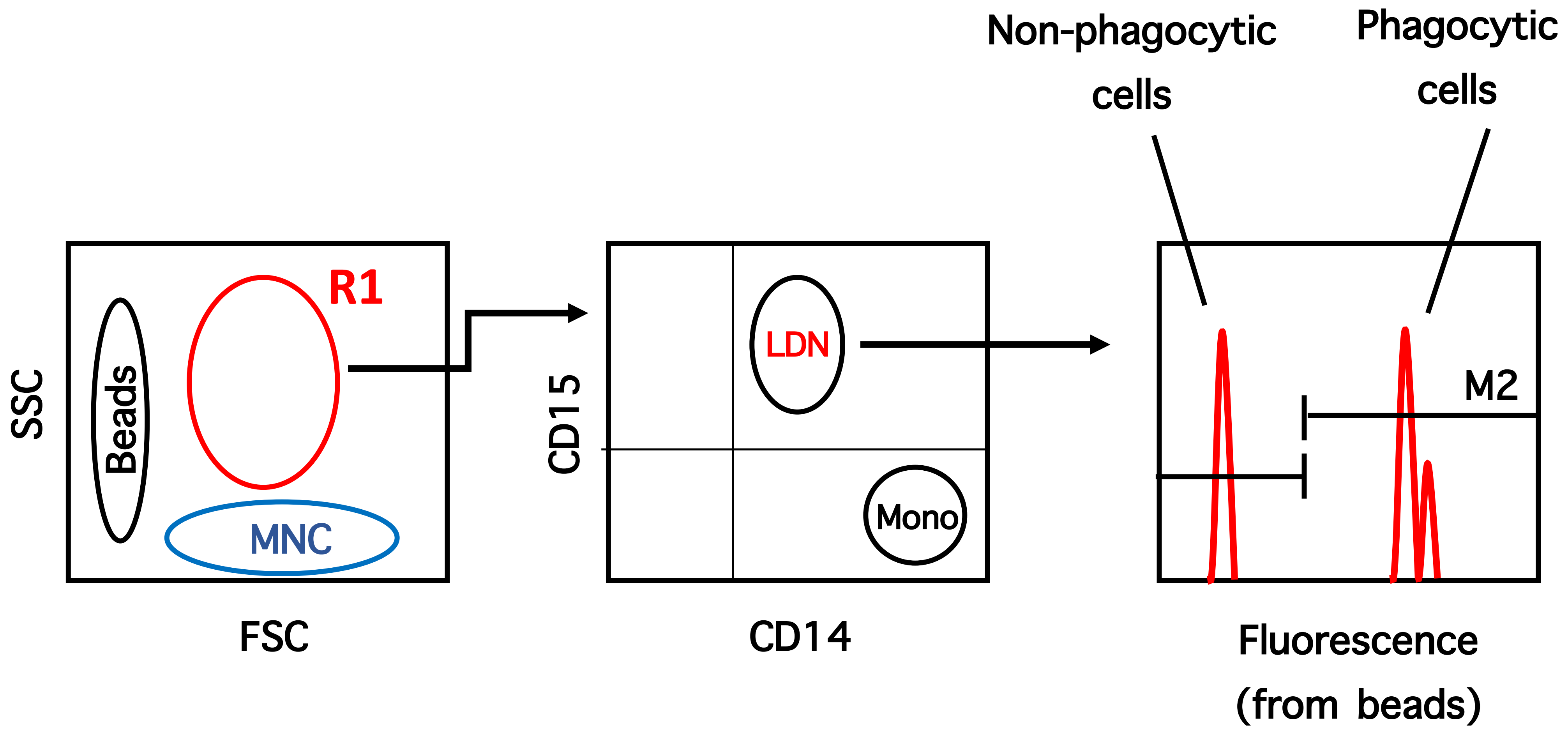

Non-phagocytic cells
Phagocytic cells
R1
Beads
MNC
SSC
FSC
LDN
Mono
CD15
CD14
M2
Fluorescence (from beads)
